# A Survey on Phytochemical Composition and Biological Activity of *Zygophyllum fabago* from Iran

**DOI:** 10.15171/apb.2017.014

**Published:** 2017-04-13

**Authors:** Saeid Yaripour, Mohammad-Reza Delnavazi, Parina Asgharian, Samira Valiyari, Saeed Tavakoli, Hossein Nazemiyeh

**Affiliations:** ^1^Department of Drug and Food Control, Faculty of Pharmacy, Tehran University of Medical Sciences, Tehran, Iran.; ^2^Department of Pharmaceutical and Food Control, Faculty of Pharmacy, Urmia University of Medical Sciences, Urmia, Iran.; ^3^Department of Pharmacognosy, Faculty of Pharmacy, Tehran University of Medical Sciences, Tehran, Iran.; ^4^Drug Applied Research Centre, Tabriz University of Medical Sciences, Tabriz, Iran.; ^5^Department of Pharmacognosy, Faculty of Pharmacy, Tabriz University of Medical Sciences, Tabriz, Iran.; ^6^Department of Medical Biotechnology, Pasteur Institute of Iran, Tehran, Iran.; ^7^ Research Center for Pharmaceutical Nanotechnology, Faculty of Pharmacy, Tabriz University of Medical Sciences, Tabriz, Iran.

**Keywords:** Zygophyllum fabago L., Zygophyllaceae, Preparative HPLC, Zygocaperoside, MTT, DPPH

## Abstract

***Purpose:*** Zygophyllum fabago L. (Z. fabago) is a widespread perennial herb which is used as a medicinal plant in traditional medicine of Iran, Turkey and China. The present study was a survey on phytochemical constituents and biological activities of this plant.

***Methods:*** Methanolic extract of the roots was fractionated over a C-18 pre-packed cartridge (Sep-pak) and chromatographic separation was performed on a reversed-phase preparative HPLC. Structural elucidation of the isolated compounds was carried out using UV, ^1^H-NMR and 13C-NMR spectral analyses. Furthermore, the chemical compositions of the essential oil of the aerial parts were identified by GC-MS analysis. Antiproliferative and antioxidant activities of all extracts from aerials were determined by MTT and DPPH assays, respectively.

***Results:*** Phytochemical investigation on the plant roots led to the isolation and identification of two the 60% methanol-water Sep-pak fraction, a prenylated flavone glycoside, 6-C-prenyl-7-O-[ β -D-4'''-O-acetyl-glucopyranosyl-(1'''→2'')-β-D-glucopyranosyl] apigenin, which was named as a Zygocaperoside and also, other flavonoid, was named as the Isorhamnetin -3-O glucoside. None of the extracts showed antiproliferative effect against cancerous cells. However, among the extracts, methanolic extract indicated antioxidant activity. Moreover, essential oils of flowers and leaves of plant have high amounts of sesquiterpene hydrocarbons and diterpenoides.

***Conclusion:*** The results of present study introduce Z. fabago roots as a new source of flavonoid glycosides and suggest it as an appropriate candidate for further pharmacological studies.

## Introduction

Medicinal plants have always been regarded as a valuable source of new bioactive lead compounds in drug development researches. *Zygophyllum fabago* L. (*Z. fabago*) (Syrian bean-caper) belonging to the family Zygophyllaceae is a perennial herbaceous plant native to southwestern and central parts of Asia, south of Europe and north of Africa.^1^ The aerial parts of *Z. fabago* have been reported internally as anti-rheumatic, anthelminthic, cathartic, anti-asthmatic, antitussive, expectorant and anti-inflammatory and externally for skin diseases, wounds, septic, and injuries.^2^ In folk medicine of Iran, this species has been named as "Qeich" with known anthelmintic and cathartic properties.^3^

The plant also known as "Memeli Uzerlik" in Azerbaijan province- Iran and its roots and aerial parts are used by indigenous Azeri people (Tabriz, Miyana, Khoy and Urmia) topically to relieve inflammatory and painful symptoms caused by insect bites (bee and scorpion). Previous pharmacological studies have shown potent butirylcholinesterase (BChE) inhibitory effects and considerable anti-fungal and anti-bacterial effects for *Z. fabago* plant.^4,5^ This species has also been considered for allergenic potential of its pollen grains.^6-8^ Profilin, a known allergen protein, has been recognized as responsible for this immunological reactions.^6-8^ Previous phytochemical investigations on the bark and aerial parts of *Z. fabago* have reported the isolation of 27-nortriterpenoid glycosides, sulphated triterpenoid saponins (fabagoin and zygophylosides E, G, O-R) and disulfated triterpenoid derivatives.^2,9-13^ Zygophylosides A, a disulphated saponin isolated from the aerial parts of *Z. fabago* has also been reported to possess a considerable inhibitory effect on Urease enzyme (87% inhibition at 0.5 mM).^2^ To the best of our knowledge, there is no report on phytochemical constituents of‏ ‏ *Z. fabago* roots and this is the first report on isolation and structure elucidation of a prenylated flavone glycoside (Zygocaperoside) from the roots of this medicinal species‏.

## Materials and Methods

### Plant Materials

The roots and aerials samples of *Z. fabago* were collected from Tabriz (East-Azarbaijan province, Iran) in July 2012. The voucher specimen of the plant was authenticated by Prof. Hossein Nazemiyeh and deposited under the code of TBZ-fph 744 at the herbarium of Faculty of pharmacy, Tabriz University of Medical Sciences (Tabriz, Iran).

### Extraction and fractionation of root parts

The air-dried and powdered roots (200 g) were Soxhlet-extracted successively with *n*-hexane, dichloromethane and methanol (1.2 L each). The obtained extracts were concentrated using a rotary evaporator at 45 °C. A portion of the methanol extract (2g × 2) was fractionated on a C-18 pre-packed cartridge (Sep-pak, 10 g, Waters) by step gradient of MeOH-H_2_O mixtures (10:90, 20:80, 40:60, 60:40, 80:20, 100:0) to get six fractions. All fractions were dried using a rotary evaporator at 45 °C.

### Isolation procedure

The (40:60) and (60:40) methanolic fractions were subjected to further phytochemical analysis using preparative reversed-phase HPLC (Shimadzu, HPLC LC-8A, SPD-M10A diode array detector, Japan). The chromatographic separation was performed on ODS Column (Dr. Maisch, 250 mm × 20 mm i.d., particle size 10 µm, Germany). The mobile phase time program was as: linear gradient of 25-40% CH_3_CN in H_2_O during 0-50 min; linear gradient of 40-55% CH_3_CN in H_2_O during 50-62 min; linear gradient of 55-25% CH_3_CN in H_2_O during 62-75 min at flow rate of 20 mL/min to get compound 1 (4.3 mg, *t*_R_ = 17.5 min) and compound 2 (16 mg, *t*_R_ = 26.2 min). The structure of isolated compounds were elucidated using UV, ^1^H-NMR and ^13^C-NMR (Bruker, Germany) spectral analyses.

### Extraction of aerial parts

The air-dried and ground leaves and flowers (100 g each) were individually macerated with methanol at room temperature. The obtained total methanolic extracts were concentrated using a rotary evaporator at 45 °C.

### Eessential oils of aerial parts

The air-dried and comminuted leaves and flowers (100 g) were separately subjected to essential oil extraction using hydrodistillation method for 4 h by a clevenger-type apparatus. The obtained essential oils were dried over anhydrous sodium sulfate and stored at 4 °C protected from light until analysis.

### GC/MS analysis

GC/MS analysis of the oil was performed on an Agilent HP-6890 gas chromatograph (Agilent Technologies, CA, USA) with a HP-5MS 5% phenyl methyl siloxane capillary column (30 m **×**0.25 mm, 0.25 µm film thickness; Restek, Bellefonte, PA) equipped with an Agilent HP-5973 mass selective detector in the electron impact mode (Ionization energy: 70 eV). Oven temperature was kept at 60 ºC for 3 min initially, and then raised at the rate of 3 °C/min to 250 °C. The temperatures of injector and detector were set at 220 and 290 ºC, respectively. The flow rate of Helium (as carrier gas) was 1 ml/min. Aliquots of 1.0 µl of diluted samples (1/1000 in n-pentane, v/v) were injected manually into the system. The quantitative analyses data were obtained by calculation of peaks area percent. The retention indices (RI) of the compounds were calculated by injecting the homologous series of n-alkanes in conditions equal to the samples. The compositions of the essential oils were identified using computer matching with the Wiley7n.L library, and also by comparison of the retention indices and fragmentation patterns of the mass spectra with data published in the literature.

### DPPH free radical-scavenging assay 

Free radical-scavenging potentials of the methanolic extracts of leaves, flowers and roots were evaluated using 2, 2-diphenyl-1-picrylhydrazyl (DPPH) method. In brief, 2 ml of freshly prepared sample solutions (10 mg/ml) were serially diluted with methanol to get concentrations of 0.5 to 3.125×10^-2^ mg/ml. 2 ml of DPPH (Sigma) solution (80 μg/ml in methanol) was then added to diluted solutions. The obtained solutions were kept 30 min at 25 °C and protected from light for any reaction to take place. Then, absorbance were recorded at 517nm. Butylated hydroxytoluene (BHT) was used as a positive control.

### *In vitro* cytotoxic activity assay

Three tumor cell lines, MCF-7 (human breast adenocarcinoma), A-549 (non-small cell line carcinoma) and HT-29 (human colon adenocarcinoma) and a normal cell line, MDBK (Madin-Darby bovine kidney) were purchased from Pasture Institute of Iran, Tehran, Iran. The cell lines were cultured in Dulbecco's Modified Eagle's Medium (DMEM) supplemented with 10% fetal bovine serum (FBS) and 1% penicillin-streptomycin in a 5% CO_2_ incubator at 37°C. In vitro cytotoxic activities of the extracts of leaves and flowers were evaluated by MTT (3-(4, 5-dimethylthiazol-2-yl)-2, 5-diphenyltetrazolium bromide) colorimetric assay. Cells were seeded into 96-well plates at a density of 0.5-1.5 ×10^4^ cells/well and incubated for 24 h at 37°C. The medium was then replaced by fresh medium containing different concentrations of extracts and incubated for 72 h at 37°C. After that, the medium was replaced by fresh medium containing MTT and incubated for additional 4 h. During this period, MTT is reduced to formazan (purple dye) by living cells. Finally, the precipitated formazan crystals were dissolved in DMSO (200 µl) and absorbance was recorded at 570 nm, using a TECAN microplate reader. Cytotoxic activities of the extracts were defined as the concentrations causing a 50% reduction in viability of cells relative to the negative control which was exposed to the solvent without extract.

## Results

**Figure 1:**
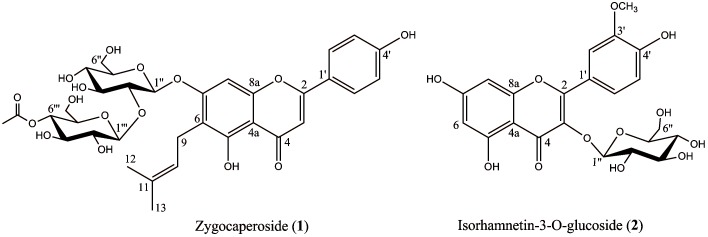
Chemical structures of compound 1 and 2 from the roots of *Z. fabago*

This study was planned to the isolation of 2 compounds from the roots of *Z. fabago,* Zygocaperoside and Isorhamnetin-3-O glycoside (Figure 1). The chemical structure of isolated compounds were elucidated unequivocally through UV and NMR and also all spectroscopic data were in agreement with respective published data.^14-21^ The data of ^1^H-NMR and ^13^C-NMR of the compounds are shown in Table 1 and Table 2.

### Spectroscopic data of compound 1

Yellow solid; On-line UV (λ max): 260, 264, 268 and 310 nm; ^1^H-NMR (200 MHz, CD_3_OD, δ/ppm, J/Hz). ^1^H and ^13^C-NMR data are shown in Table 1.

### Spectroscopic data of compound 2

^1^H and ^13^C-NMR data are shown in Table 2.

### Essential oil compositions

The hydrodistillation of *Z. fabago* leaves gave yellowish oil with a yield 0.1% (v/w), on dry weight basis. Five compounds representing 95.7% of the oil were identified as a result of GC/MS analysis of the leaves essential oil (Table 3). The results showed that the oil was rich in phytol (62.1%), a diterpenoid, as the main compound. Two non-terpenes, namely β-damascenone (12.6%), and β-ionone (15.9%) were also found in high amounts in leaves oil. Hydrodistillation of the flowers also afforded pale yellow oil (yield 0.1% (v/w)). GC/MS analysis of the obtained essential oil resulted in identification of fifteen compounds, of which ar-curcumene (20.5%), caryophyllene oxide (10.9%), espathulenol (10.2%) and bicyclogermacrene (8.8%) were the most abundant components (Table 4). Neophytadiene with the relative percentage of 4.2% was also identified as a diterpenoid present in the essential oil of plant flowers.

**Table 1:** NMR spectroscopic data of compound 1

**Position**	**δ**_C_	**δ**_H_ ** (** ***J*** **)**	**Position**	**δ**_C_	**δ**_H_ **(** ***J*** **)**
2	165.47	-	1"	100.79	5.07,(d,8.4)
3	106.82	6.59, s	2"	83.22	*
4	183.37	-	3"	74.69	*
5	152.93	-	4"	70.46	*
6	116.02	-	5"	76.45	*
7	172.08	-	6"	64.03	*
8	102.80	6.70, s	1'"	103.55	4.54
8a	150.78	-	2'"	72.03	*
4a	101.57	-	3'"	67.33	*
9 prenyl	22.76	overlapped	4'"	71.55	*
10	122.90	5.36, *br t*	5'"	72.41	*
11	128.68	-	6'"	63.77	*
12	19.95	1.34, *s*	-	-	-
13	25.01	1.29, *s*	-	-	-
1'	122.13	-	-	-	-
2'	128.86	7.89,(*d*,8.4)	-	-	-
3'	116.02	6.94,(*d*,8.4)	-	-	-
4'	161.87	-	-	-	-
5'	116.02	6.94,(*d*,8.4)	-	-	-
6'	128.86	7.89,(*d*,8.4)	-	-	-
OCO-CH3	20.01	2.19, *s*	-	-	-
OCO-CH3	176.82	-	-	-	-

^1^H (200MHz) and ^13^C (50MHz) in CD_3_OD, δ in ppm,* J* in Hz) ; * overlapping signals in 3.5-4.5ppm.

**Table 2:** NMR spectroscopic data of compound 2

**Position**	**δ**_C_	**δ**_H_ ** (** ***J*** **)**	**Position**	**δ**_C_	**δ**_H_ ** (** ***J*** **)**
2	156.99	-	1"	102.67	5.34,(d,6)
3	134.17	-	2"	71.79	*
4	177.83	-	3"	73.61	*
4a	104.24	-	4"	70.08	*
5	161.49	-	5"	76.54	*
6	98.68	6.22, (*d*, 2)	6"	67.32	*
7	164.96	-	-	-	-
8	93.61	6.43, (d, 2)	-	-	-
8a	161.49	-	-	-	-
1'	121.48	-	-	-	-
2'	112.92	8.04, (d, 2)	-	-	-
3'	146.99	-	-	-	--
4'	149.55	-	-	-	-
5'	114.69	6.95, (d, 8)	-	-	-
6'	122.37	7.66,(dd,8.2)	-	-	-
OCH_3_	55.44	3.99, s	-	-	-

^1^H (200MHz) and ^13^C (50MHz) in CD_3_OD, δ in ppm,* J* in Hz) ; * overlapping signals in 3.5-4.5ppm.

**Table 3:** Chemical composition of the leaves essential oil of *Z. fabago*.

**No.**	**Compound**	**%**	**RI**
1	β-Damascenone	12.6	1383
2	β-Ionone	15.9	1487
3	Megastigmatrienone	1.6	1585
4	Hexahydroxyfarnesyl acetone (Phytone)	5.1	1857
5	Phytol	62.1	1942
	Oxygenated sesquiterpenes	5.1	-
	Diterpenes	62.1	-
	Non-terpenes	28.5	-
	Total identified	95.7	-

RI: Relative retention indices to C8-C24 n-alkanes on HP-5MS column.

**Table 4:** Chemical composition of the flowers essential oil of *Z. fabago*.

**No.**	**Compound**	**%**	**RI**
1	α-Citral	2.3	1338
2	β-Damascenone	1.4	1383
3	E-Caryophyllene	7.3	1417
4	Alloaromadendrene	0.9	1462
5	ar-Curcumene	20.5	1479
6	β-Ionone	4.6	1487
7	α-Zingiberene	2.9	1493
8	Bicyclogermacrene	8.8	1500
9	δ-Cadinene	1.5	1522
10	Espatulenol	10.2	1577
11	Caryophyllene oxide	10.9	1582
12	Bicyclo[4.4.0]dec-1-ene, 2-isopropyl-5-methyl-9-methylene-	3.8	1653
13	Hexadecanoic acid	7.7	1959
14	Neophytadiene	4.2	2014
15	Pentacosane	4.6	2500
	Oxygenated monoterpenes	2.3	-
	Sesquiterpene hydrocarbons	50.7	-
	Oxygenated sesquiterpenes	21.1	-
	Diterpenes	4.2	-
	Non-terpenes	8.2	-
	Total identified	86.5	-

RI: Relative retention indices to C_8_-C_24_ n-alkanes on HP-5MS column.

### Antioxidant activity

The reduction in the absorption intensity of methanol solutions of DPPH radical in the presence of antioxidants at 517 nm is usually used asa measure of antioxidant activity. The ability of a sample to scavenge DPPH radical was determined on the base of its concentrations providing 50% inhibition (IC_50_). In this experiment, IC_50_ values of the total methanolic extracts of leaves, flowers and roots were obtained 0.24, 0.20‏ and 0.39 mg/ml, respectively. The IC_50_ value of Butylated HydroxyToluene (BHT) was 0.02 mg/ml. In comparison to BHT (as a powerful antioxidant), the extracts of leaves and flowers of *Z. fabago* showed the remarkable results in free radical-scavenging activity.

### Cytotoxic activity of aerial parts of plant

The results of cytotoxic activity of extracts of leaves and flowers are shown in Table 5.

Based on these results, it is indicated that both of leaves and flowers extracts had low cytotoxic activities (IC_50_>100 µg/ml) on cancerous cell lines in comparison to the literature data for IC_50_ values of cytotoxic materials.

**Table 5:** The IC_50_ values (µg/ml) obtained from MTT assay

-	**MDBK**	**A-549**	**MCF-7**	**HT-29**
Leaves extract	>100	>100	99.0	>100
Flowers extract	>100	>100	>100	>100

## Discussion

The preparative HPLC of fraction C (40% and 60% MeOH-H_2_O Sep-pak fraction) resulted in the isolation of two flavonoid glycoside. The structure of isolated compounds was studied by UV, ^1^H-NMR and ^13^C-NMR spectral analyses.

The UV spectrum of compound 1 showed a series of peaks at 260, 264, 268, 278(sh) and 310 nm characteristic for a flavone derivative. ^1^H-NMR spectrum revealed two symmetrical doublet resonance at δ 7.89 and 6.94 (*J*= 8.4 Hz) representing *para*-substituted B ring (AA'BB' system). A singlet resonance at δ 6.59 was also assigned for H-3. Three aliphatic resonances at δ 5.36 (1H, *br t*), 1.29 (3H, *s*) and 1.34 (3H, *s*) suggested the presence of a prenyl group which was supported by the ^13^C-NMR spectrum of compound. The expected doublet resonance of two protons of prenyl group at δ 3.4 (2H, overlapped) was obscured by sugar protons signals. The downfield shift of C-8 from δ 93.4 to 102.8 indicated the connection of sugar moiety to OH-7 of flavone. Prenyl group to C-6 of flavone derivative was proved by a downfield shift of C-6 from 99.40 to 116.02 ppm.^14^ Inspection of ^13^C-NMR spectrum displayed two glucopyranosyl units from anomeric resonances at δ 100.79 and 103.55 as well as other ten resonances at δ 63.77-83.22 ppm. Assignment of two anomeric resonances at δ 5.07 (1H, *d*, 8.0) and 4.55 (1H, *d*) and twelve multiple resonances at 3.1-4.4 ppm in ^1^H-NMR spectrum also confirmed the presence of two glucopyranosyl units in the structure of isolated compound. Comparison of the ^1^H- and ^13^C-NMR data with those reported in literature resulted to identification of β -D-glucopyranosyl-(1→2)-β-D-glucopyranosyl as a disaccharide moiety of the isolated flavone glycoside.^15-17^One methyl singlet at δ 2.19 with a carbonyl resonance in δ 176.82 was assigned for one acetyl moiety in ^1^H and ^13^C-NMR spectra of the isolated compound. Comparison of the ^13^C-NMR spectral data of disaccharide moiety with those reported in literature revealed the downfield shifts of C-4''', and upfield shifts for C-3''' and C-5'''. On the basis of this evidence, the acetyl group is located at C-4''' of the sugar moiety.

Consequently, the structure of isolated compound was elucidated as 6-C-prenyl-7-O-[ β -D-4'''-O-acetyl-glucopyranosyl-(1'''→2'')-β-D-glucopyranosyl] apigenin, a new compound which was named as Zygocaperoside (Figure 1).

^1^H-NMR spectrum of compound 2, revealed a doublet resonance at δ 8.04 (1H, *d*, 2‏.0) was specified for H-2'. The doublets at 6.95 (*J*= 8 Hz) and 7.66 (*J*= 8 Hz) indicated ortho-coupled aromatic H-atoms assignable to H (5') and H (6'), respectively. In addition, doublet resonances at δ 6.22 (1H, 2.0) and δ 6.43 (1H, 2.0) for the H (6) and H (8), indicated the meta coupled connection. Furthermore, some peaks at δ 3-4 ppm showed the presence of glucose at C-3. The assignment of all ^13^C-NMR signals were confirmed by comparing with the published data.^18-21^ Among the volatile compounds diterpenoids and Sesquiterpene hydrocarbons were high amounts in leaves and flowers respectively. In the other words, the amounts of Oxygenated sesquiterpenes in the leaves and the flowers considerably are different. Furthermore, free-radical-scavenging activity of the corresponding extracts was evaluated *in vitro* by the DPPH assay. The DPPH-scavenging capacity of the extracts was compared with known antioxidants, BHT as a positive control. Among the extracts, the MeOH extract showed the most potent free-radical-scavenging activity with a RC_50_ value of 0.39 mg/mL which could be attributed to the presence of the isolated flavones exhibited potent antioxidant activities in the various studies.^22,23^ Both DCM and n-Hexane extracts showed low potency in this assay; this may be explained by deficiency of hydrogen donating components. The anti-proliferative property of the aerial parts of extracts has been evaluated by the MTT assay.^24^ None of the extracts showed significant effect against cancerous cells. It is also indicated that there was no obvious cytotoxic effect on non-cancerous cell lines (MDBK). It seems that aerial parts of *Z. fabago* are not the first choices for further evaluations in cancer researches but it is suggested to isolate and purify the compounds from the aerial parts of the plant which would be have cytotoxic effects on cancerous cell lines without cytotoxicity on non-cancerous cell line.

## Conclusion

Flavonoids have considered for their various health benefits such as antioxidant, hepatoprotective, anti-inflammatory, anticancer, antibacterial and antiviral activities. It has also been reported that prenylation enhances the antibacterial, anti-inflammatory, antioxidant, cytotoxicity, larvicidal and estrogenic activity of the flavonoids.^16,17^ The result of present study on isolation and identification of a prenylated flavon-O- glucoside (compound 1) and Isorhamnetin-3-O glucoside (compound 2) from *Z. fabago* roots is indicative of more medicinal potentials of this species and suggests it as an appropriate candidate for further biological and pharmacological studies.

## Ethical Issues

Not applicable.

## Conflict of Interest

The authors have no conflicts of interest to declare.
